# Improved Quality of Life Among Chronic Pancreatitis Patients Undergoing Total Pancreatectomy With Islet Autotransplantation—Single Center Experience With Large Cohort of Patients

**DOI:** 10.3389/ti.2023.11409

**Published:** 2023-09-04

**Authors:** Mariagrazia Coluzzi, Morihito Takita, Giovanna Saracino, Abdul Rub Hakim Mohammed, Carly M. Darden, Giuliano Testa, Ernest Beecherl, Nicholas Onaca, Bashoo Naziruddin

**Affiliations:** ^1^ Simmons Transplant Institute, Baylor University Medical Center, Dallas, TX, United States; ^2^ Unit of General and Emergency Surgery, Azienda Ospedaliera Regionale San Carlo, Potenza, Italy; ^3^ Department of Radiation Health Management, Fukushima Medical University, Fukushima, Japan; ^4^ Baylor Scott and White Research Institute, Dallas, TX, United States; ^5^ LifeGift, Fort Worth, TX, United States

**Keywords:** pancreatitis, islet autograft, quality of life, pain, diabetes mellitus

## Abstract

Total pancreatectomy with islet autotransplantation (TPIAT) is the treatment of choice to preserve pancreatic endocrine function, alleviate pain, and improve quality of life (QoL) when other strategies are ineffective for chronic pancreatitis (CP) patients. This study utilized pancreatic disease-specific surveys developed by the European Organisation for Research and Treatment of Cancer (EORTC) to conduct a comprehensive, single-center examination of a large cohort of patients to gain understanding of QoL post-TPIAT. Two validated QoL surveys of the EORTC—QLQ-C30 and QLQ-PAN26—were administered in a prospective cohort of CP patients during pre-and post-operative scheduled visits. A total of 116 patients responded to the preoperative survey and were included in this study. The global health scale of QLQ-C30 was significantly improved after TPIAT when compared to baseline with delta scores of 24.26, 20.54, and 26.7 at 1, 2, and 3 years post-TPIAT (*p* < 0.001). The EORTC-PAN26 revealed significant improvements in symptom scales for pancreatic pain, bloating, digestive symptoms, taste, indigestion, weight loss, body image, and future worries. The comprehensive surveys in such a large cohort expands the QoL criterion in CP patients and indicates significant improvement in QoL post-TPIAT, further validating TPIAT as a treatment option for refractory CP.

## Graphical Abstract

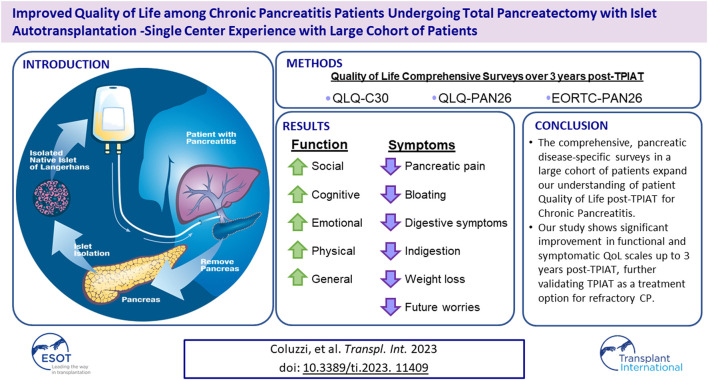

## Introduction

Chronic pancreatitis (CP) is an irreversible inflammatory and fibrotic disease of the pancreas leading to varying degrees of exocrine and endocrine dysfunction. In severe cases, CP can lead to permanent loss of exocrine and endocrine function [1]. Furthermore, 75% of CP patients experience abdominal pain which can become debilitating [2]. CP patients report recurrent hospitalizations and numerous treatments to relieve pain and restore some semblance of normality in their quality of life (QoL). Initial medical management for CP may include but is not limited to narcotic analgesics, replacement of pancreatic enzymes, and radiological endoscopic procedures [3–5]. Patients with progressive symptoms in which medication and endoscopic intervention fails may be candidates for surgery [6]. Surgical techniques such as Puestow, Frey, Beger, and Whipple procedures are performed to achieve pain relief in CP patients. However, there is no evidence that these procedures lead to stable maintenance of endocrine function [7].

Total pancreatectomy followed by islet autotransplantation (TPIAT) is a preferred technique to preserve endocrine function and alleviate pain when other strategies are ineffective [8]. The first human TPIAT was performed by Dr. David Sutherland at the University of Minnesota in 1977. The rationale for this procedure is by removing the source of pain and disease exacerbations, this will improve a very poor QoL, reduce or eliminate chronic narcotic use, and facilitate return to work and self-care [9].

During the last 30 years, numerous collaborations between various North American centers, including ours, have developed the TPIAT program and documented metabolic outcomes. Many studies have reported achieving the main objective, improvement of QoL, through the SF-36 questionnaire, which evaluates health-related QoL [10–15]. In the current study, we evaluated QoL in patients who received TPIAT for CP at Baylor University Medical Center (Dallas, TX, United States) using the European Organisation for Research and Treatment of Cancer (EORTC) Quality of Life Questionnaire (QLQ)-C30 survey combined with the QLQ-PAN26, designed specifically for patients with pancreatic disease [16].

## Patients and Methods

### Study Participants

This prospective observational study assessed the patient-oriented outcomes of QoL in CP patients who underwent TPIAT at Baylor University Medical Center. All patients were evaluated by a multidisciplinary team and had multiple indications for TPIAT. Patient eligibility for TPIAT includes intractable pain despite previous medical treatment, detectable endogenous insulin secretion capacity evident by serum C-peptide, and the capacity to consent to the treatment. Pregnant women were not eligible for the surgical procedure. We obtained consent for the intervention and study enrollment from all participants after they had been adequately informed of the risks. This study was conducted after approval of the institutional review board of Baylor Scott and White Research Institute (IRB#009-271).

### Data Collection

Patients were asked to answer two QoL surveys of the EORTC—QLQ-C30 and QLQ-PAN26—before TPIAT and completed the survey during follow-up or by mail at 1, 2, and 3 years after transplantation. The EORTC QLQ-C30 and QLQ-PAN26 instruments were selected because they have been validated and used in trials to evaluate other pancreatic procedures [17–19]. The QLQ-C30 consists of 30 questions. The first section examines functioning: physical functioning, role functioning, emotional functioning, cognitive functioning, and social functioning in addition to a single item of global health. The second section addresses nine symptoms: nausea and vomiting, pain, fatigue, insomnia, loss of appetite, constipation, diarrhea, dyspnea, and financial difficulties. The QLQ-PAN26 assesses functioning and pancreatic-specific symptoms. It has 26 questions that evaluate pancreatic pain, bloating, digestive symptoms, taste, indigestion, flatulence, weight loss, dry mouth, hepatic symptoms, altered bowel habit, body image, trouble with side effects, future worries, and planning of activities in addition to healthcare satisfaction and sexuality [17–19]. All scales range from 0 to 100. QLQ-C30 high scores indicate healthier status or improved QoL for global health. High scores on the symptom scales correlate with a poor QoL. High scores on the QLQ-PAN26 indicate a poor QoL, except for healthcare satisfaction and sexuality.

**FIGURE 1:**
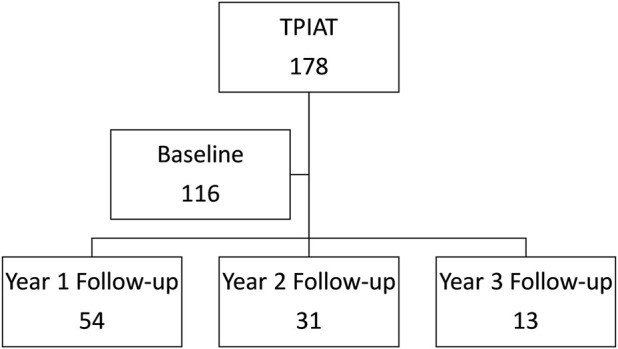
Consortium diagram of patient participation in the two surveys from baseline to 3 years follow-up.

The patients completed these surveys during their regularly scheduled follow-up appointments in electronic or hard copy format. We confirmed the validity of the electronic format. Surveys were completed via telephone or mail for 2 and 3 years post-TPIAT if the patients were unable to attend their clinic visits. Subjects with reduced ability to understand the questionnaires were excluded from the study. Details on the patient participation in these surveys is shown in Figure 1.

All clinical data were recorded for each patient in a prospectively maintained database. Preoperative and postoperative clinical data in this study included levels of hemoglobin A1c, serum C-peptide, exogenous insulin requirement, pain score based on visual analog scale (VAS), and morphine equivalent dose. The VAS for pain ranged from 0 (*no pain*) to 10 (*most severe pain*). The daily dose of opioids was converted into morphine milligram equivalents (MME).

### TPIAT Procedure

All patients underwent total pancreatectomy with the surgical technique described previously [20–23], with or without splenectomy based on surgeon decision. The distal common bile duct was removed, and the pancreatic blood supply was preserved during surgery as long as possible to minimize islet cell ischemia. On the back table, the spleen (if removed) and duodenum were detached from the pancreas, the pancreatic duct was cannulated, and the pancreas was placed in a container with cold preservation solution. Subsequently, the pancreas was transferred to a current good tissue practice (cGTP) facility for islet isolation processing.

Liberase MTF with Thermolysin MTF (Roche, Basel, Switzerland) or Collagenase NB with neutral proteases (SERVA Electrophoresis GMbH, Heidelberg, Germany) was infused into the pancreatic duct for digestion. Islets were isolated by the modified Ricordi method, which has been previously described [24, 25]. When the tissue volume (mL) exceeded 0.25 times body weight (kg), islets were purified with a COBE 2991 cell processor (Caridian BCT Inc., Lakewood, CO) using a density-adjusted iodixanol-based continuous density gradient. Endotoxin testing, gram staining, and bacterial and fungal cultures were performed on the final products as indicators of sterility. Isolated islets were infused into the portal vein via the superior mesenteric vein with heparin (70 unit/kg body weight) while the patient was under general anesthesia. The portal vein pressure was regularly monitored during the islet infusion.

### Statistical Analysis

Data were presented as numbers and percentages for binary and categorical variables or as median and interquartile range (IQR) or as mean with standard deviations (SD) for continuous variables. The primary outcomes in this study were independent trends over time of the various scales and items of the EORTC QLQ-C30 and QLQ-PAN26. The surveys were administered at four time points: baseline, 1, 2, and 3 years. Raw scores measured at baseline and at years 1 and 2 were analyzed in longitudinal repeated measures analyses.

Generalized least square models without random effects, fitted by restricted maximum likelihood (REML) were used to examine if there was a differential effect across time (baseline to follow-up) in score measurements. The analysis focused on longitudinal single group analyses, where a single homogeneous population was followed over time. To account for the correlation in repeated measurements on the same subject, using various correlation structures with constant variance were considered. The correlation structure was selected based on AIC [29].

Due to the small sample size of respondents, scores measured at year 3 were not considered in longitudinal analyses. Additionally, a generalized least square model without random effects with restricted maximum likelihood (REML) was used instead of ordinary maximum likelihood estimation (MLE) [26, 27].

The constant variance assumption was checked using typical residual plots. The univariate normality assumption was checked using typical Q-Q plots on residuals. For checking the correlation pattern, variograms based on estimating correlations of all possible pairs of residuals at different time points were used [26–30].

Delta, defined as a difference over time from baseline, was assessed to estimate clinical significance in the EORTC questionnaires, according to recommendations by Osoba, where a difference in health-related QoL score of 10 points or more is regarded as clinically significant [31, 32]. The radar charts depict patients’ scores for the EORTC QLQ-C30 and EORTC QLQ-PAN26 scales for each domain as observed marginal means at different time points. Each domain is represented on separate axes (scaled from 0 to 100). All statistical analyses were conducted using R Statistical Software (version 4.1.2; R Foundation for Statistical Computing, Vienna, Austria). For the longitudinal analyses, the gls function from the nlme: Linear and Non-linear Mixed Effects Models, R package version 3.1-162, and the rms: Regression Modeling Strategies, R package version 6.7-0, developed by Harrell [28] were used.

## Results

### Participant Characteristics

Between 31 March 2011, and 1 April 2021, 178 consecutive patients underwent TPIAT at our center. Among that group, 116 patients agreed to answer the two QoL surveys before transplant (65% participation rate) and were included in this study. The demographic and clinical characteristics of the study participants are presented in Table 1. Participants’ median age at TPIAT was 41.1 (*30.4–49.0*) years, 35% were male, and the median body mass index was 26.3 (21.5–29.8) kg/m^2^. To better understand our patient cohort disease progression, we looked at prior pancreatic interventions. Prior endoscopic stent management failed for 82 patients (71%). 18 patients (16%) had previous pancreatic surgery before TPIAT. Within this cohort, the median duration of diagnosed pancreatitis was 5.0 (3.0, 10.0) years. Post-TPIAT data revealed a median transplanted islet equivalent dose was 5.1 (2.9–7.2) × 10^3^ IEQ/kg patient body weight. The median follow-up was 78.8 months (range 9.4–125.5 months) and at 1, 2, and 3 year follow-up, 2, 6, and 9 patients had died, respectively.

**TABLE 1:** Characteristics of study participants.

Characteristics	Overall (*n* = 116)
Age (years): median (*IQR*)	41.1 (*30.4*, *49.0*)
Male: *n* (%)	41 (35%)
Body mass index (kg/m^2^): median (*IQR*)	26.3 (*21.5*, *29.8*)
Epidemiology: *n* (%)	
Alcoholic	9 (7.8%)
Autoimmune	7 (6.0%)
Hereditary	19 (16%)
Idiopathic	55 (47%)
Other	26 (22%)
Pancreatic duct stent insertion or EST: *n* (%)	82 (71%)
Past history of pancreas operation: *n* (%)	18 (16%)
Duration of symptoms (years): median (*IQR*)	5.0 (*3.0*, *10.0*)
Dose (×10^3^ IEQ/kg patient body weight): median (*IQR*)	5.07 (*2.93*, *7.15*)

EST, endoscopic sphincterotomy; IEQ, islet equivalent. IQR values are in parentheses and italicized.

### Metabolic Outcomes and Pain Control Status

**FIGURE 2:**
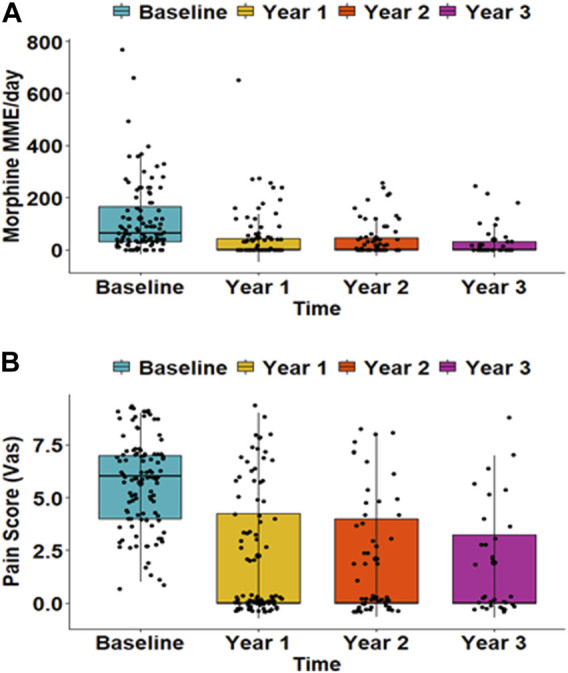
**(A)** Morphine equivalent dose and **(B)** pain scores at baseline and at 1, 2, and 3 years after total pancreatectomy followed by islet autotransplantation. The morphine dose is presented as daily milligrams morphine equivalent (MME). Pain scores were evaluated with a visual analog scale (VAS) that ranged from 0 (no pain) to 10 (the most severe). Both daily morphine dose and pain score were significantly reduced over time (*p <* 0.001).

12.1% of patients had diabetes before TPIAT, and 78%, 73%, and 71% were insulin-dependent at 1, 2, and 3 years after TPIAT, respectively. Glycemic outcomes pre- and post-TPIAT are outlined in Table 2. Daily morphine requirements and pain scores significantly decreased over time after TPIAT (*p* < 0.001) (Figure 2). There was notable decrease in mean MME dose with 118 (±*137*) mg before TPIAT and 44 (±*93*), 42 (±*68*), and 35 (±*65*) mg at years 1, 2, and 3, respectively. Pain scores evaluated with VAS also decreased after TPIAT: 5.7 (±*2.1*) at baseline, 2.2 (±*2.9*) at year 1, 2.1 (±*2.8*) at year 2, and 1.9 (±*2.6*) at year 3.

**TABLE 2:** Metabolic and pain outcomes at baseline and after total pancreatectomy followed by islet autotransplantation.

Variables	Baseline (*n* = 116)	Follow-up		
		Year 1 (*n* = 79)	Year 2 (*n* = 40)	Year 3 (*n* = 27)
Endocrine outcomes				
Hemoglobin A1c (%)	6.0 (*1.1*)	7.3 (*2.0*)	7.3 (*2.4*)	7.0 (*1.4*)
Serum C-peptide (ng/dL)	1.8 (*1.3*)	1.2 (*1.2*)	1.4 (*1.5*)	1.1 (*1.3*)
Fasting blood glucose (mg/dL)	102 (*29*)	152 (*94*)	151 (*65*)	124 (*54*)
Exogenous insulin amount (unit/day)	2.2 (*8.0*)	14.7 (*15.0*)	15.5 (*15.9*)	14.4 (*17.5*)
Pain control				
Pain score^a^	5.7 (*2.1*)	2.2 (*2.9*)	2.1 (*2.8*)	1.9 (*2.6*)
Morphine equivalent dose (mg/day)	118 (*137*)	44 (*93*)	42 (*68*)	35 (*65*)

^a^ Evaluated with the visual analog scale, ranging from 0 (no pain) to 10 (the most severe pain). SD values are in parentheses and italicized.

### EORTC QLQ-30 and QLQ-PAN26 Surveys

**FIGURE 3:**
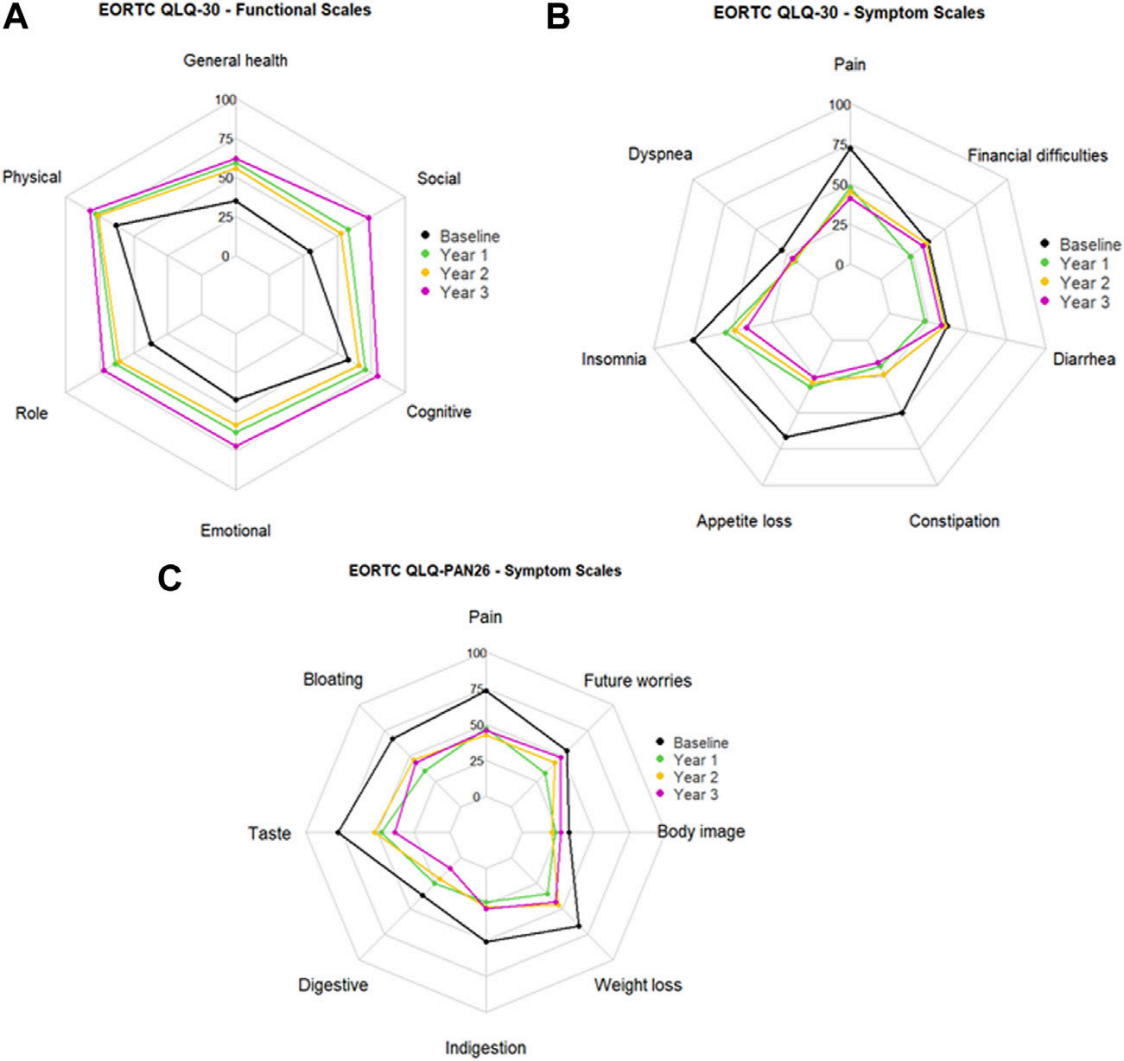
Mean scores at baseline and 1, 2, and 3 years after total pancreatectomy followed by islet autotransplantation for **(A)** EORTC QLQ-C30 functioning scale, **(B)** EORTC QLQ-C30 symptom scale, and **(C)** EORTC QLQ-PAN26 symptom scale.

Initially, 116 patients responded to the preoperative survey and respondents decreased at yearly follow-up. 54 patients completed the survey at year 1, 31 patients at year 2, and 13 patients at year 3. Radar charts visually display each domain of the EORTC QLQ-C30 functional scales and symptom scales of EORTC QLQ-C30 and EORTC QLQ-PAN26 (Figure 3). We displayed domains with a statistically significant trends over time as indicated by generalized least square models for repeated measures.

EORTC QLQ-30 survey functional scores increase with improved QoL after 1, 2, and 3 years (Figure 3A). In our patient cohort, the generalized least square models for repeated measures of functioning scales demonstrated that global health QoL, physical functioning, role functioning, emotional functioning, cognitive functioning, and social functioning significantly increased over 2 years post-TPIAT (*p* < 0.001, <0.001, <0.001, <0.001, = 0.007, and <0.001, respectively). Delta mean scores outlined in Table 3 indicate the change from baseline (pre-TPIAT) to each follow-up year. In each functional scale domain of EORTC QLQ-C30 the score was ≥10 points, indicating a clinically relevant improvement from baseline.

**TABLE 3:** Responses on the EORTC QLQ-C30 survey before and after total pancreatectomy followed by islet autotransplantation.

Domain	Baseline (*n* = 116)	Follow-up			Delta			*p-*value
		1 year (*n* = 54)	2 years (*n* = 31)	3 years (*n* = 13)	Delta 1 year	Delta 2 years	Delta 3 years	
Global health	34.84 (*23.79*)	59.10 (*27.82*)	55.38 (*27.01*)	61.54 (*26.69*)	24.26	20.54	26.7	<0.001
Functional scales								
Physical functioning	62.82 (*24.14*)	78.27 (*22.92*)	76.13 (*21.83*)	82.05 (*24.70*)	15.45	13.31	19.23	<0.001
Role functioning	37.21 (*30.72*)	63.58 (*36.78*)	60.75 (*38.38*)	71.79 (*32.19*)	26.37	23.54	34.58	<0.001
Emotional functioning	42.17 (*26.36*)	62.81 (*31.75*)	58.06 (*30.99*)	71.79 (*20.84*)	20.64	15.89	29.62	<0.001
Cognitive functioning	55.26 (*29.94*)	70.37 (*31.67*)	65.59 (*27.87*)	79.49 (*18.20*)	15.11	10.33	24.23	0.007
Social functioning	30.32 (*32.65*)	58.33 (*36.02*)	52.69 (*36.03*)	73.08 (*33.71*)	28.01	22.37	42.76	<0.001
Symptom scales								
Fatigue	72.41 (*26.39*)	47.59 (*31.61*)	45.16 (*24.83*)	41.03 (*20.98*)	−24.82	−27.25	−31.38	<0.001
Nausea and vomiting	57.90 (*33.08*)	28.70 (*31.79*)	35.48 (*35.42*)	24.36 (*29.36*)	−29.2	−22.42	−33.54	<0.001
Pain	72.41 (*26.39*)	47.59 (*31.61*)	45.16 (*24.83*)	41.03 (*20.98*)	−24.82	−27.25	−31.38	<0.001
Dyspnea	29.02 (*31.25*)	18.52 (*27.22*)	19.35 (*25.49*)	20.51 (*28.99*)	−10.5	−9.67	−8.51	0.0697
Insomnia	75.00 (*30.10*)	54.32 (*36.22*)	48.39 (*32.02*)	41.03 (*33.76*)	−20.68	−26.61	−33.97	<0.001
Appetite loss	66.95 (*34.19*)	32.10 (*35.44*)	29.03 (*31.90*)	25.64 (*33.76*)	−34.85	−37.92	−41.31	<0.001
Constipation	50.00 (*37.94*)	17.90 (*26.47*)	23.66 (*27.48*)	15.38 (*22.01*)	−32.1	−26.34	−34.62	<0.001
Diarrhea	37.07 (*35.10*)	22.84 (*31.61*)	35.48 (*38.43*)	33.33 (*38.49*)	−14.23	−1.59	−3.74	0.061
Financial difficulties	37.07 (*35.10*)	22.84 (*31.61*)	35.48 (*38.43*)	33.33 (*38.49*)	−14.23	−1.59	−3.74	0.061

SD values are in parentheses and italicized.

Lower symptom scores in the EORTC QLQ-C30 indicate better QoL (Figure 3B). The generalized least square model of the symptom scales revealed that fatigue, nausea and vomiting, pain, insomnia, appetite loss, and constipation were significantly reduced post-TPIAT (*p* < 0.001, <0.001, <0.001, <0.001, = 0.001, and <0.001, respectively). Moreover, the corresponding delta scores showed changes of ≥20 points, indicating that the reduction in these symptoms was also clinically meaningful (Table 3).

EORTC QLQ-PAN26 surveyed symptom scales pre- and post- TPIAT in which lower scores indicate better QoL (Figure 3C). Again, the generalized least square model demonstrated that pancreatic pain, bloating, digestive symptoms, taste, indigestion, weight loss, body image, and future worries had a statistically significant trend of reduction over time (*p* < 0.001, <0.001, <0.001, = 0.009, = 0.001, <0.001, = 0.003, and = 0.009, respectively). The corresponding delta scores indicated clinically meaningful reductions in symptoms in all domains except flatulence, hepatic symptoms, and trouble with side effects (Table 4). Functional scales in QLQ-PAN26 related to satisfaction with healthcare and sexuality were also significantly ameliorated after TPIAT (*p* = 0.004 and <0.001, respectively).

**TABLE 4:** Responses on the EORTC QLQ-PAN26 survey before and after total pancreatectomy followed by islet autotransplantation.

Domain	Baseline (*n* = 116)	Follow-up			Delta			*p-*value
		1 year (*n* = 54)	2 years (*n* = 31)	3 years (*n* = 13)	Delta 1 year	Delta 2 years	Delta 3 years	
Symptom scales								
Pancreatic pain	73.41 (*24.95*)	47.07 (*30.98*)	42.74 (*23.25*)	45.51 (*26.27*)	−26.34	−30.67	−27.90	<0.001
Bloating	66.67 (*30.46*)	35.19 (*32.64*)	46.24 (*32.97*)	43.59 (*31.58*)	−31.48	−20.43	−23.08	<0.001
Digestive symptoms	77.97 (*28.66*)	47.53 (*33.71*)	52.15 (*34.09*)	38.46 (*32.19*)	−30.44	−25.82	−39.51	<0.001
Taste	37.07 (*33.99*)	25.31 (*33.61*)	20.43 (*28.12*)	10.26 (*21.01*)	−11.76	−16.64	−26.81	0.009
Indigestion	50.86 (*36.11*)	23.46 (*27.19*)	26.88 (*29.08*)	28.21 (*35.61*)	−27.40	−23.98	−22.65	<0.001
Flatulence	47.41 (*34.09*)	48.15 (*37.01*)	50.54 (*38.37*)	64.10 (*34.59*)	0.74	3.13	16.69	−0.868
Weight loss	66.67 (*30.46*)	35.19 (*32.64*)	46.24 (*32.97*)	43.59 (*31.58*)	−31.48	−20.43	−23.08	<0.001
Dry mouth	42.53 (*34.78*)	30.25 (*30.56*)	38.71 (*39.53*)	20.51 (*25.60*)	−12.28	−3.82	−22.02	0.057
Hepatic symptoms	17.98 (*19.35*)	16.98 (*23.01*)	17.20 (*22.56*)	17.95 (*19.79*)	−1.00	−0.78	−0.03	−0.9524
Altered bowel habit	37.36 (*30.03*)	41.67 (*28.18*)	47.31 (*35.25*)	50.00 (*34.02*)	4.31	9.95	12.64	0.2427
Body image	33.05 (*22.84*)	22.84 (*24.29*)	20.97 (*18.24*)	26.92 (*31.58*)	−10.21	−12.08	−6.13	0.004
Troubled with side effects	7.76 (*22.14*)	9.26 (*20.90*)	8.60 (*22.72*)	15.38 (*32.25*)	1.50	0.84	7.62	−0.914
Future worries	54.89 (*37.37*)	33.33 (*31.72*)	43.01 (*30.05*)	48.72 (*39.94*)	−21.56	−11.88	−6.17	<0.001
Planning of activities	56.03 (*34.50*)	41.36 (*32.33*)	48.39 (*34.25*)	51.28 (*32.25*)	−14.67	−7.64	−4.75	0.03
Functional scale								
Satisfaction with healthcare	18.25 (*23.97*)	29.94 (*31.62*)	22.58 (*23.78*)	30.77 (*28.74*)	11.69	4.33	12.52	0.004
Sexuality	78.26 (*24.50*)	51.23 (*33.93*)	57.53 (*30.98*)	44.87 (*32.90*)	−27.03	−20.73	−33.39	<0.001

SD values are in parentheses and italicized.

To safeguard against potential bias in study findings, an analysis was conducted to examine whether study patients who completed at least a baseline questionnaire and excluded patients that never participated in the study, displayed distinct characteristics worth exploring (Table 5). Moreover, a comparison between patients who responded at baseline only with participants who responses at follow-up surveys was conducted follow-up and shown in Table 6. Pearson’s Chi-squared test; Wilcoxon rank sum test; Fisher’s exact test were used for group comparisons. Both study participants and excluded patients exhibited similar characteristics unlikely to lead to potential bias with a strong impact on the study findings. The results showed that participants who did not return follow-up surveys had a significantly higher body mass index (BMI) (*p* = 0.036) with a median of 27.7, (IQR 23.6–32.0), compared to participants with follow-up measures, who had a median of 25.2 (IQR: 20.7–28.9).

**TABLE 5:** Characteristics of study participants and non-participants.

Characteristics	Study participants (*n* = 116)	Excluded participants (*n* = 62)	*p*-value
Age (years): median (*IQR*)	41.1 (*30.4*, *49.0*)	39.2 (*28.6*, *49.6*)	0.680
Male: *n* (%)	41 (35%)	25 (40%)	0.510
Body mass index (kg/m^2^): median (*IQR*)	26.3 (*21.5*, *29.8*)	25.1 (*21.6*, *29.6*)	0.620
Epidemiology: *n* (%)			0.087
Alcoholic	9 (7.8%)	3 (4.8%)	
Autoimmune	7 (6.0%)	1 (1.6%)	
Hereditary	19 (16.4%)	21 (33.9%)	
Idiopathic	55 (47.4%)	23 (37.1%)	
Other	26 (22.4%)	14 (22.6%)	
Pancreatic duct stent insertion or EST: *n* (%)	82 (71%)	42 (69%)	0.820
Past history of pancreas operation: *n* (%)	15 (13%)	10 (16%)	0.560
Duration of symptoms (years): median (*IQR*)	5.0 (*4.0*, *8.0*)	5.0 (*3.0*, *10.0*)	0.820
Dose (×10^3^ IEQ/kg patient body weight): median (*IQR*)	5.07 (*2.93*, *7.15*)	4.47 (*2.88*, *6.12*)	0.440

EST, endoscopic sphincterotomy; IEQ, islet equivalent. IQR values are in parentheses and italicized.

**TABLE 6:** Characteristics of study participants by follow-up group.

Characteristics	With follow-up (*n* = 55)	No follow-up (*n* = 61)	*p*-value
Age (years): median (*IQR*)	37.7 (*30.8*, *48.4*)	42.7 (*30.4*, *49.0*)	0.580
Male: *n* (%)	18 (33%)	23 (38%)	0.420
Body mass index (kg/m^2^): median (*IQR*)	27.8 (*23.6*, *32.0*)	25.5 (*20.7*, *28.9*)	0.036
Epidemiology: *n* (%)			0.087
Alcoholic	1 (1.8%)	8 (13%)	
Autoimmune	2 (3.6%)	5 (8.2%)	
Hereditary	12 (22%)	7 (11%)	
Idiopathic	26 (47%)	29 (48%)	
Other	14 (25%)	12 (25%)	
Pancreatic duct stent insertion or EST: *n* (%)	39 (71%)	43 (70%)	0.960
Past history of pancreas operation: *n* (%)	11 (20%)	7 (11%)	0.210
Duration of symptoms (years): median (*IQR*)	5.0 (*4.0*, *10.0*)	5.0 (*3.0*, *10.0*)	0.380
Dose (×10^3^ IEQ/kg patient body weight): median (*IQR*)	5.43 (*3.76*, *6.95*)	4.63 (*2.74*, *7.34*)	0.440

EST, endoscopic sphincterotomy; IEQ, islet equivalent. IQR values are in parentheses and italicized.

## Discussion

In North America, multiple centers perform TPIAT and have demonstrated an improvement in health-related QoL in patients with CP and recurrent acute pancreatitis. The objective means used to ascertain an improvement in QoL were the SF-36 and SF-12 questionnaires [10–15, 33]. These studies evaluated QoL by reporting scores for body pain, mental composite, physical composite, and social functioning, and results showed persistent improvement for up to 5–10 years follow-up [10, 13]. By implementing pancreatic disease-specific surveys EORTC QLQ-30 and QLQ-PAN16, we were able to gain a better understanding of the QoL of our TPIAT patients by including more specific criterion in the surveys. These surveys were originally used to evaluate patients with pancreatic cancer and were validated in 2005 for evaluation of pancreatic surgery for CP [16, 18, 34, 35]. EORTC QLQ-C30 global general health scores increased by delta score of 26.70 (77% increase) which further validates that TPIAT improves patient general health.

Management of pain is a major objective of TPIAT and thus another critical metric for success. Nonetheless, pancreatic pain can be misinterpreted or reported generically through the use of the VAS score and the *Body Pain* scale in the SF-36. We utilized surveys with more specific measures to more accurately present statistical and clinical evidence of persistent pain reduction for up to 3 years. Pancreatic pain evaluated with the QLQ-PAN26 revealed clinical and statistical improvement with a delta score of 27.90. It is noteworthy that 71% of patients had a previous pancreatic duct stent inserted and 16% had a previous pancreas operation with neither resolving pain maintenance. The survey showed an average percent reduction in morphine dose of approximately 37%, 35%, and 30% at 1, 2, and 3 years, respectively, with mean daily MME decreasing from 118 (±*137*) mg at baseline to 35 (±*65*) mg at 3 years. Our results are consistent with the international consensus guideline, where opioid doses were reduced by 71%, 69%, and 67% at 1, 2, and 5 years [36]. Our study supports improved pain management and metabolic functioning even in patients with a history of pancreatic surgery.

In addition to the pain reduction of TPIAT, we observed a significant reduction of other symptoms of CP including nausea, vomiting, weight loss, and digestive disturbances. Our study found no statistical variation in diarrhea symptoms, but there was an overall decreasing trend. This is similar to Crosby et al. report in which more than 60% of patients still reported diarrhea after TPIAT, adding that enzyme non-adherence was not a major contributor [37]. Our surveys showed no improvement in flatulence and altered bowel habits. These symptoms may be related to exocrine insufficiency, intestinal resection with reconstruction in TPIAT, and new intestinal motility after the reduction of narcotic drugs [38, 39]. As the TPIAT procedure involves infusion of pancreatic islets into the portal vein of the liver, it was important for us to document any changes in hepatic symptoms which were not present in our cohort.

A limitation of this study was reduced sample size in follow-up years as patient participation waned. We have observed reports of similar instances in other centers [10, 13, 28, 40, 41]. However, we were able to evaluate the same patient sample over time and have statistically significant data that allows us to highlight new aspects of QoL in TPIAT.

In conclusion, we found that pancreatic disease-specific surveys allow us to gain a deeper understanding of patient QoL post-TPIAT for CP. We observed significant improvements in QoL after TPIAT, and expanded our knowledge in the functional and symptom scales for CP patients up to 3 years post-transplant. Our study strongly supports the benefits of TPIAT as a treatment option for refractory CP.

## Data Availability

The original contributions presented in the study are included in the article/supplementary material, further inquiries can be directed to the corresponding author.

## Abbreviations

CP, chronic pancreatitis; EORTC, European organisation for research and treatment of cancer; MME, morphine milligram equivalents; QoL, quality of life; SD, standard deviation; TPIAT, total pancreatectomy followed by islet autotransplantation; VAS, visual analog scale.
